# Ghrelin Levels and Decreased Kidney Function in Patients with Early Stages of Chronic Kidney Disease Against the Background of Obesity

**DOI:** 10.25122/jml-2020-0152

**Published:** 2020

**Authors:** Nataliia Vasylivna Gubina, Iryna Hryhorivna Kupnovytska, Vasyl Hryhorovych Mishchuk, Halyna Dmytrivna Markiv

**Affiliations:** 1. Department of Clinical Pharmacology and Pharmacotherapy, Ivano-Frankivsk National Medical University of Ministry of Health of Ukraine, Ivano-Frankivsk City, Ukraine; 2. Communal Non-profit Enterprise “Central City Clinical Hospital” of Ivano-Frankivsk City Council, Ivano-Frankivsk City, Ukraine

**Keywords:** Chronic kidney disease, obesity, cystatin C, ghrelin

## Abstract

The importance of kidney damage in obese patients is due to the increasing incidence of nephropathies associated with metabolic disorders, their predisposition to a progressive course of the disease, and the need to optimize early disease detection.

The purpose of our work is to study the level of cystatin C, ghrelin, and their interrelation in patients with early stages (I-II) of chronic kidney disease (CKD) against the background of obesity.

The indicators of daily microalbuminuria in patients of both groups were studied, and it was found that in patients with stage 2 CKD with obesity, it was 1.2 times higher than in patients with stage 1 CKD with obesity. Patients of the second group revealed a direct middle correlation between daily albuminuria and body mass index (BMI), which indicates deterioration in the functional state of the kidneys against the background of obesity.

We obtained an increase in the serum concentration of cystatin C in stage 2 CKD with obesity, reflecting the state of glomerular filtration and the degree of renal function decrease. Correlation analysis showed a positive relation of cystatin C with increased urinary albumin excretion in both groups, plasma creatinine content, BMI, and age.

Simultaneously with the increase in the level of cystatin C and the decrease in the glomerular filtration rate, there was an increase in ghrelin levels in stage 2 CKD and the progression of obesity, while such dependence was not seen in stage 1 CKD.

## Introduction

In recent years, there has been an increase in the incidence of chronic kidney disease (CKD) due to an increase in the number of patients with metabolic disorders such as obesity, type 2 diabetes, and hyperuricemia [1]. Arterial hypertension, as well as an increase in the consumption of non-steroidal anti-inflammatory drugs, make a significant contribution to the development of such comorbidity [2].

The urgency of kidney damage in obese patients is due to the increasing incidence of nephropathy associated with metabolic disorders, their tendency to a progressive course of the disease, and the need to optimize early disease detection [3]. After 5 years of follow-up of obese patients without diabetes, according to Tozawa *et al.*, 5.7% of patients developed CKD [4]. There is currently no consensus on the mechanisms of glomerulopathy, hyperfiltration and albuminuria in obese patients [5]. Mathew *et al.* found a faster decrease in glomerular filtration rate due to increased blood pressure after studying the relation between the components of the metabolic syndrome and the progression of CKD [6]. On the other hand, in a study evaluating the results of kidney biopsy from normal-weight donors and overweight donors, the latter had a significantly larger glomerular surface area, but no signs of glomerulosclerosis were found [7]. It is also believed that the rate of progression of CKD with obesity depends on the body mass index (BMI) [8].

In recent decades, there has been an understanding that CKD is not only a consequence of various diseases but also a predictor of the progression of certain cardiovascular diseases and mortality, the combination of which is constantly growing [9]. The growing role of CKD in this tandem is partly due to the widespread use of glomerular filtration rate (GFR) screening techniques to establish the CKD stage [10]. At the same time, most calculation formulas based on the amount of endogenous creatinine, including its clearance, in particular the D.W. Cockroft and M.H. Gault formula, MDRD equation, D.E. Salazar – G.B. Corcoran method, were found to be unsuitable for the assessment of GFR in obese or overweight people [11]. In this regard, a method using cystatin C provides additional opportunities to diagnose the early stages of CKD [12].

Serum cystatin C (sCysC) is a low-molecular-weight protein belonging to the family of cysteine proteinase inhibitors that play an important role in the intrinsic cellular metabolism of various peptides [13]. It is synthesized at a constant rate by all nuclear cells, freely filtered by glomeruli, completely reabsorbed, and metabolized in the kidney tubules [14]. In some studies, it has been shown that cystatin C, as an endogenous marker of the GFR, compared with creatinine in some patient populations, is ahead of the latter regarding sensitivity and specificity of renal function control [15]. It has been established that an increase of cystatin C in blood serum is observed with a decrease in glomerular filtration, and an increase in its excretion in the urine indicates dysfunction of the cells of the proximal renal tubules [16]. Increased serum cystatin C levels with normal creatinine levels indicate a subclinical kidney disease associated with a high risk of developing a clinically significant stage of kidney disease, leading to the early development of cardiovascular complications [17].

In recent years, ghrelin, leptin, and insulin have played an important role in the occurrence of obesity and its impact on renal function and pathology [18]. Ghrelin is a hormone consisting of 28 amino acids and is a ligand of the growth hormone receptor and is mainly produced by the X/A-like cells of the gastric mucosa. In addition, ghrelin has a cardioprotective effect in ischemia, enhances vasodilation, and is involved in the regulation of blood pressure [19]. Among the various mechanisms of action of ghrelin, there are stimulation of differentiation of preadipocytes into adipocytes, inhibition of lipolysis in vitro, enhancement of lipogenesis in the bone marrow [20]. Currently, ghrelin remains the only known centrally acting orexigenic hormone, which is produced peripherally, secreted into the bloodstream mainly from gastric endocrine cells, and can be synthesized in the kidneys [21]. At the same time, ghrelin helps maintain a balance between vasoconstrictors (endothelin 1) and vasodilator (NO) compounds, inhibits cellular apoptosis in the cardiovascular system in patients with metabolic syndrome [22]. In healthy people, ghrelin is metabolized and excreted by the kidneys and plays an important role in the pathogenesis of protein balance changes, inflammation and cardiovascular complications in CKD [23]. According to some authors, when renal function changes, the level of ghrelin is variable, and its secretion can be affected by both the central signals of the hypothalamus and the metabolic status of peripheral tissues [24]. Along with impaired general and local metabolism, ghrelin levels may be affected by decreased renal clearance [25].

Currently, studies have been conducted mainly on changes in ghrelin levels in end-stage chronic kidney disease [26], and there is little work that studied its changes in patients with its early stages with obesity.

The purpose of the work was to study the level of cystatin C, ghrelin and their interaction in patients with early (I-II) stages of chronic kidney disease against the background of obesity.

## Material and Methods

One hundred fifty-eight patients with chronic kidney disease who were hospitalized at the department of Arterial Hypertension of the Communal Non-profit Enterprise “Ivano-Frankivsk Regional Clinical Cardiological Centre of Ivano-Frankivsk Regional Council”, Urological and Cardiology Department of the Communal Non-profit Enterprise “Central City Clinical Hospital of Ivano-Frankivsk City Council” in Ivano-Frankivsk City were examined. Of them, 67 are women, and 91 are men. The average age of the examined patients was: 55.36 ± 2.02 years in women and 47.45 ± 2.66 years in men. Stage 1 CKD was diagnosed in 70 patients (31 women, 39 men; the mean age was 46.43 ± 3.77 years), stage 2 CKD was diagnosed in 88 patients (36 women, 52 men; the average age was 53.07 ± 2.61 years). The reasons for the development of CKD were as follows: infections of the upper urinary tract in 11.8% cases, urolithiasis in 19.89% cases, 15.76% suffered from glomerulonephritis with symptomatic renal parenchymal hypertension, abnormalities in the development of the urine-reproductive system in 6.9% cases, hypertension in 29.1% cases and coronary heart disease with heart failure in 18.89% cases. The average duration of CKD was 7.1 years. Exclusion criteria were diabetes mellitus, hypothalamic and endocrine obesity, acute myocardial infarction, congestive heart failure IIB – III according to the Vasylenko-Strazhesko system and III-IV according to the New York Heart Association (NYHA) classification, liver failure, stages 3-5 CKD. The control group consisted of 10 apparently healthy individuals (3 women and 7 men), whose average age was 36.7 ± 8.6 years. All patients underwent general clinical examinations. The CKD stage was established according to the 2012 Kidney Disease: Improving Global Outcomes (KDIGO) guidelines [27] and the guidelines of the Institute of Nephrology of the National Academy of Medical Sciences of Ukraine. BMI was calculated using the Kettle formula (kg/m2): BMI = body weight, kg/(height), m2. In this regard, patients were divided into two groups: 1-a (70) – patients with stage 1 CKD and obesity, 2 – a (88) – patients with stage 2 CKD and obesity.

Determination of albumin in daily urine was performed using the turbidimetric method with the “Microalbumin” diagnostic kit (Germany) and evaluated in mg/day. Glomerular filtration rate was calculated according to the CRD-EPI formulas based on the level of creatinine, cystatin C and their combination (CRD-EPIcr, CRD-EPIcys, CRD-EPIcysC/cr, respectively) (ml/min/1.73 m2 for all formulas) using the US National Kidney Foundation calculator (http://www.kidney.org/professionals/kdoqi/gfr_calculator.cfm). Cystatin C level in serum (normally 0.79 – 2.15 mg/l) was examined by enzyme-linked immunosorbent assay using the Human Cystatin C ELISA (Czech Republic) kit on the STAT FAX analyzer (No. 7898). Ghrelin level in serum (pg/ml) was determined by enzyme-linked immunosorbent assay using the Human Ghrelin EIA (CIF) kit on the STAT FAX analyzer.

The study protocol was approved by the Ethics Committee of the Ivano-Frankivsk National Medical University, protocol No. 97/17 of 19 October 2017. All patients gave informed consent to participate in the study. The study was conducted in accordance with the principles of the Helsinki Declaration of the World Medical Association “Ethical principles for medical research involving human subjects” of 01 October 2008, No. 900_005.

Statistical analysis of the results was performed using the statistical software package Statistica 6.0 using Student’s t-test. The correlation was assessed by Spearman’s rank correlation coefficient. The discrepancy of the results at p<0.05 was considered statistically significant.

## Results

Urinary albumin excretion is currently considered to be a marker of endothelial dysfunction and atherosclerosis; therefore, it is advisable to determine this indicator in both CKD and metabolic disorders. According to the results of our studies, the level of daily microalbuminuria in patients of groups 1 and 2 exceeded the indicators in healthy people by 7.4 and 8.9 times, respectively (p1,2<0.001). Comparing the indicators of daily microalbuminuria in patients of both groups, it was found that in patients with stage 2 CKD against the background of obesity, it was 1.2 times higher (p>0.05) than in patients with stage CKD on the background of obesity (Table 1). In patients of group 2, a direct average correlative relation was found between daily albuminuria and BMI-r = 0.56, p<0.05, which indicates a deterioration of the functional state of the kidneys on the background of obesity.

**Table 1:** Characteristics of laboratory parameters in patients with CKD of stages 1 and 2 and obesity.

Indicator	Healthy, n=10	Group 1, n=70	Group 2, n=88
**Age, years**	36.7 ± 8.6	46.43 ± 3.77	53.07 ± 2.61
**BMI, kg/m^2^**	21.44 ± 0.39	34.15 ± 1.1	35.59 ± 0.82
**Albuminuria. mg/day**	19.0 ± 0.96	141.1 ± 12.66*	170.05 ± 17.6*
**Cystatin C, mg/l**	0.78 ± 0.02	1.43 ± 0.13*	1.79 ± 0.13*
Creatinine, umol/l	77.71 ± 1.48	94.26 ± 2.32*	105.03 ± 1.56*°
**GFR, ml/min/1.73 m^2^** CRD-EPIcr	104.67 ± 1.36	97.3 ± 1.52*	73.04 ± 1.58*°
**GFR, ml/min/1.73 m** ^2^ CRD-EPIcys	101.16 ± 1.86	94.56 ± 1.34*	67.78 ± 2.05*°
**GFR, ml/min/1.73 m**^2^ CRD-EPIcysC/cr	103.3 ± 1.27	98.0 ± 2.76	64.5 ± 2.07*°
Ghrelin, pg/ml	39.3 ± 2.1	15.4 ± 1.1*	56.3 ± 1.7*°

*- consistent when compared with healthy; ° - consistent when comparing groups 1 and 2.

The level of cystatin C in patients of both groups also exceeded the respective indicator in healthy people – 1.8 and 2.29 times, respectively (p1,2<0.001). In patients of group 2, cystatin C was 1.25 times higher than those in group 1 (p>0.05). The increase in cystatin C serum levels reflects the state of glomerular filtration and the degree of decrease in renal function [28]. According to the results of correlation analysis, a positive relation between cystatin C and increased excretion of albumin with the urine in both groups (r1 = 0.63 and r2 = 0.56, p>0.05), the content of creatinine in blood plasma (r1 = 0, 98, p<0.001), r2 = 0.51, p<0.05) with BMI (r1 = 0.36 (p>0.05) and r2 = 0.69, p<0.05) and age (r1 = 0.55 and r2 = 0.98, p<0.05) was noted.

The glomerular filtration rate calculated based on cystatin C in patients of group 1 was slightly lower (94.56 ± 1.34) than the one calculated based on creatinine (97.3 ± 1.52), and there was no significant difference in this indicator when calculated by the CRD-EPIcys and CRD-EPIcysC/cr formulas. In patients of group 2, the glomerular filtration rate calculated based on cystatin C was significantly lower than that based on creatinine (p <0.01). The glomerular filtration rate calculated by the formula CRD-EPIcysC/cr indicates a decrease in this indicator in patients of group 2 compared with group 1 by 1.5 times (p <0.001), which confirms renal dysfunction, despite the normal values of creatinine. Reverse causality of glomerular filtration rate, calculated by the CRD-EPIcys formula, with serum cystatin C level was determined in both groups (r1= -0.49 and r2 = -0.68, p1.2<0.05). The level of creatinine and glomerular filtration rate calculated by the CRD-EPIcr formula correlated with BMI. However, the BMI-creatinine relationship was inconsistent (r1 = 0.31, r2 = 0.66). A medium negative correlation between GFR-CRD-EPIcys and BMI-r1 = -0.52 in patients of group 1 and a strong one – r2 = -0.71 in patients of group 2 (p1,2 <0.05) was determined.

The study of total ghrelin level in the serum of patients with stage 1 CKD on the background of obesity indicates a consistent (p <0.01) decrease of 2.5 times. On the contrary, the total ghrelin level in those examined for stage 2 CKD was 1.4 times higher (p <0.05). After performing correlation analysis between cystatin C and ghrelin levels, a direct correlation was found (r = 0.67, t = 2.5, p <0.05) between the increase in both indicators in patients with stage 2 CKD on the background of obesity. No correlation was found in those examined for stage 1 CKD with obesity. Decreased glomerular filtration rate in patients with stage 2 CKD and obesity had a reverse average correlation (r = -0.66, t = 3.05, p <0.05), while in patients with stage 1 CKD with obesity, these indicators were not interrelated. Other researchers point to a positive correlation between elevated ghrelin and creatinine levels in higher-stage CKD, which developed on the background of obesity, elevated blood pressure, and metabolic syndrome [29].

## Discussion

The issue of kidney damage on the background of obesity, and the associated frequency of nephropathy, is relevant today, and early diagnosis of concomitant pathology is based on the identification and assessment of pathogenetic risk factors for their formation. According to Pop *et al.*, the increase in the number of obese patients correlates with the frequency of kidney damage [30]. Among the main factors in the progression of kidney damage in obesity are insulin resistance, dyslipidemia, disorders of systemic and renal hemodynamics, renal tissue ischemia, the action of adipose tissue hormones. Identifying CKD risk factors is important, especially considering such a global problem as obesity. As indicated by Tsujimoto *et al.*, the main therapeutic goal of obesity treatment is the control and treatment of related complications, including renal pathology [31]. Microalbuminuria is a proven, highly sensitive marker of prognostic adverse kidney damage, and it also reflects the presence of endothelial dysfunction. Increased urinary albumin excretion in abdominal obesity is considered a consequence of the “organotoxic” influence of hormones produced by adipocytes [32]. Conversely, an increase in the plasma concentration of adiponectin, which counteracts the adverse effects of hyperleptinemia and hyperinsulinemia, reduces the probability of albuminuria [33]. Analysis of a group of Copenhagen residents (658 people) aged 50-89 showed that the presence of microalbuminuria indicates a high mortality rate and the development of major cardiovascular complications in the first 5 years [34]. In most cases, the early stages of CKD (1-2) are often asymptomatic, and albuminuria may be the only symptom of CKD. Such changes are especially common in kidney damage with hypertension, obesity and diabetes, complicating the diagnosis of renal dysfunction in the early stages.

Therefore, in recent years, various early specific biomarkers of kidney damage have been studied, among which much attention is paid to cystatin C. In 2005, Deo *et al.*, having compared serum creatinine and cystatin C levels with GFR, found that cystatin C is a more accurate marker of renal function damage than creatinine [35]. In 2004, the Food and Drug Administration (FDA) approved the determination of cystatin C as a marker of alternative GFR calculation for early diagnosis of renal glomerular dysfunction, determination of CKD stage, and prognosis of disease progression. Studies by the Multi-Ethnic Study of Atherosclerosis (MESA) (2014) and Cardiovascular Health Study (CHS) (2009), which included 11,909 people with CKD, showed that cystatin C, not creatinine, and GFR indicators calculated based on it play an important prognostic role in the identification of individuals with a high risk of vascular complications [36]. Having examined 4663 people over the age of 65, Shlipak *et al.* found that individuals with high levels of cystatin C without CKD anamnesis had an increased risk of adverse cardiovascular events, and 50% of them were likely to die [37]. In the Atherosclerosis Risk in Communities (ARIC) prospective study from 2012 it was found that even a minimal increase in eGFRcys (75-89 ml/min) on the background of moderate albuminuria was associated with an increased risk of coronary heart disease, heart failure, CKD and possible involvement of the peptide in the systemic immunoinflammatory response [38]. It is known that a significant part of cystatin C is synthesized by adipocytes of subcutaneous and visceral adipose tissue. The presence of obesity is associated with a significant increase in cystatin production by adipocytes [39]. Although cystatin did not seem to be dependent on body weight, age, or sex in early studies, the Third National Health and Nutrition Examination Study (NHANES III ) from 2008 showed an increase in cystatin C levels together with an increase in body weight and age [40], which to some extent explains its increase in stage 2 CKD determined by us.

Ghrelin is known to be a circulating hunger hormone, but there is very little data on ghrelin levels in non-dialysis patients with early-stage CKD [41]. The authors found that obesity was most common in stage 2 CKD. An increase in fasting ghrelin levels, which was increased 1.4 times in the examined patients, stimulates the appetite and obesity and probably leads to kidney damage, i.e., a vicious circle is created. According to the authors mentioned above, this dependence disappeared in stages 4-5 CKD. A high level of ghrelin at these stages can probably be explained by global metabolic changes, protein-energy deterioration, and chronic inflammation [42]. The authors also confirm that a decrease in glomerular filtration rate may lead to an increase in ghrelin levels. It is a higher GFR in stage 1 CKD that can explain the lower level of ghrelin in the blood of these patients.

## Conclusion

Thus, as can be seen from our study, increased ghrelin level has different meanings at different stages of chronic kidney disease. In particular, in stage 2 CKD, its growth is associated with an increase in cystatin C levels and a decrease in glomerular filtration rate and probably the progression of obesity, while in stage 1, such a relationship is not yet formed. Such changes require new approaches to the treatment of this combined pathology.

## Conflict of Interest

The authors declare that there is no conflict of interest.
